# Individual differences in effects of stressful life events on childhood ADHD: genetic, neural, and familial contributions

**DOI:** 10.1111/jcpp.70074

**Published:** 2025-11-08

**Authors:** Seung Yun Choi, Jinwoo Lee, Junghoon Park, Eunji Lee, Bo‐Gyeom Kim, Gakyung Kim, Yoonjung Yoonie Joo, Jiook Cha

**Affiliations:** ^1^ Department of Brain and Cognitive Sciences Seoul National University Seoul Korea; ^2^ Department of Psychology Seoul National University Seoul Korea; ^3^ Department of Psychology University of California San Diego San Diego CA USA; ^4^ Interdisciplinary Program in Artificial Intelligence, College of Engineering Seoul National University Seoul Korea; ^5^ Department of Digital Health, Samsung Advanced Institute for Health Sciences & Technology Sungkyunkwan University Seoul Korea; ^6^ Samsung Genome Institute, Samsung Medical Center Seoul Korea

**Keywords:** Early‐life stress, ADHD, gene‐brain‐environment, vulnerability, individual differences

## Abstract

**Background:**

This study elucidates the intricate relationship between stressful life events and the development of ADHD symptoms in children, acknowledging the considerable variability in individual responses. By examining these differences, we aim to uncover the unique combinations of factors contributing to varying levels of vulnerability and resilience among children.

**Methods:**

Utilizing longitudinal data from the Adolescent Brain Cognitive Development study (baseline: *N* = 6,303, age = 9.9), we applied Generalized Random Forest (GRF) to model the nonlinear relationships among genetic predispositions, brain features, and environmental factors.

**Results:**

Significant individual variability was observed in children's ADHD symptoms post‐stress, particularly at the 1‐year and 2‐year follow‐ups. At the 1‐year follow‐up, increased vulnerability was indicated by heightened parental mental health problems and a lower polygenic risk score for smoking. By the 2‐year follow‐up, escalated parental mental health disorders, higher ADHD polygenic risk scores (PRS), and altered structural connectivity in the cognitive control network were significant contributors to individual differences.

**Conclusions:**

These findings underscore the importance of integrating environmental, genetic, and neural variables to identify children vulnerable or resilient to developing ADHD symptoms following early‐life stress. This study demonstrates how multimodal data combined with nonparametric machine learning can advance precision psychology and psychiatry, aiding targeted support for affected children.

## Introduction

The nexus between early‐life stress and an elevated risk for developing ADHD has been consistently documented (Greeson et al., 2014; Humphreys et al., 2015, 2019). Early‐life stress refers to exposure during childhood to significant adverse events such as disaster, violence, or severe accident. Such experiences have been linked to disruptions in brain development, particularly in regions involved in cognitive control, emotional regulation, and attention processing – functions commonly impaired in ADHD (Boodoo, Lagman, Jairath, & Baweja, 2022; Carrion & Wong, 2012; McLaughlin et al., 2014). Children exposed to stress may also experience heightened physiological arousal, altered reward sensitivity, and impairments in executive function, all of which may contribute to the emergence of ADHD symptoms (Greeson et al., 2014; Martínez, Prada, Satler, Tavares, & Tomaz, 2016). Despite this established connection, the question of why only certain children develop ADHD in response to early‐life stress while others remain resilient still persists. The joint contribution of neural, genetic, and environmental factors during childhood – a critical period of significant brain development – may account for this inter‐subject variability in ADHD outcomes (Bouchard Jr. & McGue, 2003; Gilmore, Knickmeyer, & Gao, 2018; Halperin & Healey, 2011; Marsh, Gerber, & Peterson, 2008; Parasuraman & Jiang, 2012). Research with mouse models has shown that early‐life stress can lead to enduring changes in dopaminergic neurons through glucocorticoid‐mediated epigenetic regulation, particularly when combined with specific genetic vulnerabilities (Niwa et al., 2013). These modified dopaminergic pathways, integral to brain functions such as cognitive control and motivation, may also be implicated in ADHD pathophysiology (Cools, 2016; Swanson et al., 2007). Thus, understanding this dynamic interplay is essential for comprehending the differences in how children respond to similar stressful events, with not all developing ADHD symptoms.

Literature often highlights isolated aspects of this complex association. For instance, some studies focus solely on neural influences, such as decreased cortical thickness in temporal and parietal regions, which mediate the relationship between social deprivation and increased ADHD symptoms in children (McLaughlin et al., 2014). Specific brain areas, including the temporal gyrus and internal capsule, are also linked to ADHD symptoms following stressful experiences in children (Humphreys et al., 2019). However, the influence of these neural variables on ADHD symptomatology may interact with genetic and environmental factors (Cortese, 2012; Yadav et al., 2021).

Genetic factors, often represented by polygenic risk scores (PRS), may play a crucial role in determining an individual's susceptibility to ADHD symptoms following stressful experiences by interacting with brain structure or function. ADHD PRS is associated with ADHD in children, influencing brain functional connectivity (i.e. caudate‐parietal cortex, nucleus accumbens‐occipital area) (Hermosillo et al., 2020). Total cortical gray matter volume mediates the relationship between PRS for ADHD comorbid with disruptive behavior disorder and externalizing problems in late childhood (Teeuw et al., 2023). Additionally, ADHD PRS predicts the mean fractional anisotropy (FA) of white matter regions, with this FA being significantly linked to ADHD symptom scores (Albaugh et al., 2019). These findings suggest that children's genetic predisposition may shape the brain's response to early‐life stress, thereby influencing ADHD development.

Environmental factors also contribute to children's ADHD symptoms following stress exposure by interacting with genetic and neural factors. Family dynamics, interpersonal support, and neighborhood deprivation show a significant association with ADHD symptoms in children, while these environmental stressors are also linked to gray matter volume and cortical thickness (Jeong et al., 2023). Children in more chaotic households tend to exhibit more ADHD symptoms, and their ADHD PRS significantly contributes to these chaotic environments, indicating a gene–environment interaction in ADHD development (Agnew‐Blais et al., 2022).

Examining the combined and conditional contributions of genetic predispositions, neural, and environmental factors in relation to early‐life stress and ADHD development demands an integrated approach. Overlooking this interplay may result in a skewed interpretation. We address this by employing Generalized Random Forest (GRF), a nonparametric machine‐learning method adept at modeling the complex and nonlinear relationships among these factors. GRF extends the traditional random forest algorithm by integrating parametric causal inference with statistical examination of potential confounders (Athey, Tibshirani, & Wager, 2019). The flexible and inductive approach enhances the detection of subtle heterogeneity patterns not feasible with conventional methods, where researchers preselect predictors and examine interaction effects by testing each variable individually (Shiba et al., 2021). The efficacy of GRF is supported across various studies: natural disaster on cognitive disability and mental health (Shiba et al., 2021, 2022), social disconnection on suicidality (Solomonov et al., 2023), and efficacy of therapeutic interventions (Goligher et al., 2023).

We aim to elucidate how stressful life events uniquely influence the development of ADHD symptoms in children, considering the combined and conditional contributions of neural, genetic, and environmental factors. To achieve this goal with methodological precision, it is essential to reduce heterogeneity within our treatment variable (i.e. stressful life events). Aggregating distinct stressors into a single composite could obscure meaningful mechanisms and make it difficult to disentangle whether our findings reflect genuine variation in individual responses to stress or merely stem from differences in the types of stressors. To address this, we focused on a subset of events involving indirect threat exposures – events where the child was present or a witness but not the intentional target of harm (e.g. natural disasters, accidents, witnessing violence). Prior research suggests that direct and indirect exposures differ in a range of outcomes, including neurocognitive impairments (e.g. deficits in emotion attribution and executive functioning) (Fishbein et al., 2009), patterns and severity of mental health symptoms (Price, Higa‐McMillan, Kim, & Frueh, 2013), and levels of socio‐emotional factors like anxiety, emotion expression/suppression, and self‐efficacy (Rode & Rode, 2017). By constructing a more homogeneous treatment variable, our goal was to improve construct clarity and better isolate individual differences in children's vulnerability to stress‐related risk for ADHD rather than capturing variation driven by different types of stressors. Specifically, we formulated the following hypotheses: (1) Exposure to stressful life events would be positively associated with increased ADHD symptoms over the 2‐year follow‐up period. (2) Significant individual variability would emerge in these trajectories, such that the impact of stress would depend on children's baseline profiles of genetic, familial, and neural risk. For instance, higher ADHD PRS, adverse familial factors, and alterations in brain structure or connectivity would contribute to this differential vulnerability. By identifying patterns within these multimodal factors, we seek to uncover the varying degrees of vulnerability and resilience among children exposed to stress. These insights could help researchers and clinicians identify subpopulations needing targeted interventions. Ultimately, understanding these risk factors could lead to the development of strategies designed to protect children from developing ADHD after experiencing stress.

## Methods

### Participants

**Figure 1:**
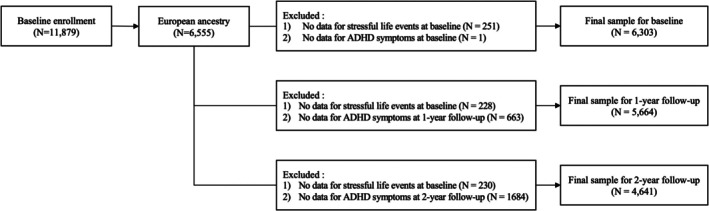
Flow chart of study sample selection

A total of 11,878 participants aged 9–10 years were drawn from the Adolescent Brain Cognitive Development (ABCD) study, a prospective longitudinal cohort study conducted across 21 sites in the United States (Data Release 4.0; http://abcdstudy.org) (Casey et al., 2018). Since our study examined the association of stressful life events and ADHD symptoms in a longitudinal design (i.e. baseline, 1‐year, and 2‐year follow‐ups), participants were excluded if they missed stressful events data at baseline, missed ADHD symptom data at each time point, or reported non‐European ancestry. To ensure consistent ancestry, we included only individuals with European ancestry, which comprised almost 80% of the sample (*N* = 6,555). This decision was based on methodological concerns about cross‐ancestry PRS applications, as most current GWAS datasets are predominantly derived from European populations. Applying European‐based PRS to individuals of different genetic backgrounds can lead to biased risk estimates and reduced predictive accuracy (Adam et al., 2023; Carlson et al., 2013; Need & Goldstein, 2009). Still, as a comparison to the main analyses, we conducted exploratory analyses including multi‐ancestry participants. Detailed procedures and results from these supplementary analyses are provided in Appendix S1 (Tables S1 and S2). We did not impute missing variables because most missing data were from non‐European ancestry, and the variables related to stressful events and ADHD symptoms were crucial for our analyses. Therefore, we opted for conservative analyses. The final sample size was 6,303 at baseline (male = 53.2%, *M*_age_ = 9.9), 5,664 at 1‐year follow‐up (male = 53.3%), and 4,641 at 2‐year follow‐up (male = 53.6%) (Figure 1). Detailed demographics are provided in Table S3.

### Measures

#### Stressful life events

The stressful life events were measured at baseline only by the Kiddie Schedule for Affective Disorders and Schizophrenia – post‐traumatic stress disorder (KSADS‐PTSD) reported by the parents (Kaufman et al., 1997), which assesses lifetime exposure to such events. In line with the study's focus on individual differences in children's vulnerability to stressful events, we constructed a treatment variable representing indirect threat exposure. Rather than aggregating heterogeneous stressors into a single composite, which could obscure interpretability, we adopted an a priori strategy to focus on a subset of events sharing key characteristics: the child was present or a witness, but not the intentional target of harm. Based on this criterion, we included eight events: natural disaster, fire, act of terrorism, war‐zone exposure, severe car accident, other significant accident requiring specialized/intensive medical care, witnessing community violence (i.e. shooting/stabbing), and witnessing domestic violence (i.e. grown‐ups push, shove, or hit each other). We excluded direct victimization and bereavement, as they represent distinct categories: direct victimization involves intentional harm directed at children, while bereavement primarily reflects psychological processes centered on grief and relational loss rather than threat‐based responses (Rubin, Malkinson, & Witztum, 2020). By maintaining this focused construct, we sought to improve the precision and specificity of our interpretations regarding individual differences in children's vulnerability to external stressors. The item was scored as 1 if the child experienced at least one event and 0 if the child did not experience any events.

#### ADHD

Children's ADHD symptoms were assessed using a parent‐reported DSM‐oriented Childhood Behavior Checklist (CBCL) (Achenbach & Edelbrock, 1991) at baseline, 1‐year, and 2‐year follow‐ups. The parents answered items on a three‐point Likert scale, with higher values indicating more ADHD symptoms. The reliability and validity of the CBCL were shown by prior studies (Biederman et al., 2001, 2021).

#### Covariates

**Figure 2:**
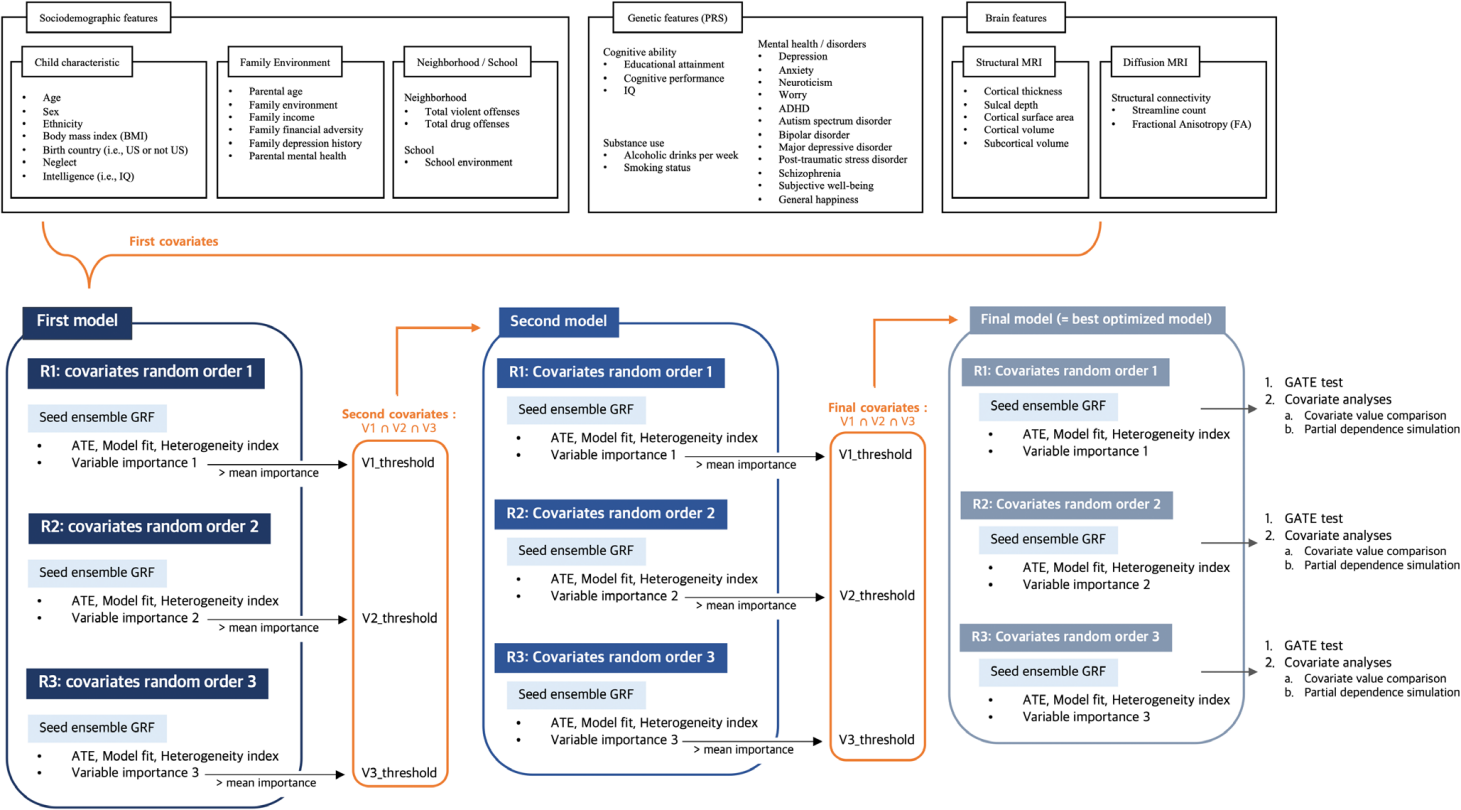
Framework of three‐step analysis in Generalized Random Forest (GRF) analysis. The three‐step analysis involved fitting the model with different covariate orders and seed ensembles to enhance robustness of the results (i.e. Random Iteration 1, 2, and 3). To find the optimal model, we progressively reduced the model size by retaining only the intersection of covariates that were above the mean importance in all three iterations. This optimization process narrowed down the covariates to those significantly contributing to individual differences, resulting in the final model. We then applied the GATE test to the final model to validate the existence of individual difference and conducted covariate analysis to identify potential risk and protective factors. ATE, average treatment effect; BMI, body mass index; GATE, group average treatment effect; PRS, polygenic risk score; R1, random iteration 1; R2, random iteration 2; R3, random iteration 3; V1, variable importance 1; V2, variable importance 2; V3, variable importance 3

We included baseline sociodemographic, genetic, and neuroimaging features (Figure 2), given their potential roles as confounders in the association between stressful events and ADHD symptoms. Variable selection was guided by theoretical relevance, as established in prior literature, and by data availability within the ABCD dataset (Appendix S2).

The sociodemographic characteristics consist of the child's characteristics (i.e. age, sex, ethnicity, BMI, birth country, abuse, neglect, and IQ) and the environment of the family, neighborhood, and school. Family environment included parental age, marital status, parental education level, income, family conflict, family financial adversity, family depression history, and parental mental health (e.g. externalizing problems, depressive problems). Neighborhood environment included rates of total violent and drug offenses in the neighborhood (United States Department of Justice, Office of Justice Programs, Federal Bureau of Investigation, 2012). The school environment evaluated the child's perception of the school climate. See Appendix S2 for a detailed description of measurements.

For genetic characterization of the participants, we utilize PRS of 17 complex traits as genetic features: educational attainment, cognitive performance, IQ, depression, anxiety, neuroticism, worry, ADHD, autism spectrum disorder, bipolar disorder, major depressive disorder, PTSD, schizophrenia, subjective well‐being, general happiness, alcoholic drinks per week, and smoking status (Joo et al., 2024). These PRSs were derived using publicly accessible genome‐wide association studies (GWAS) summary statistics (Appendix S2, Table S4).

The brain morphometric features (i.e. surface area, thickness, sulcal depth, and volume) and diffusion measures (i.e. FA and streamline count) collected at baseline were included in the analyses. T1‐weighted 3D structural images (1‐mm voxel resolution) were acquired using either a 3T Siemens, Phillips, or General Electric scanner with a 32‐channel head coil. We applied ABCD quality control conditions to exclude low‐quality brain images. Cortical and subcortical estimates were extracted using FreeSurfer 7.1.1 (https://surfer.nmr.mgh.harvard.edu). Cortical regions were labeled with the Desikan‐Killiany atlas (Desikan et al., 2006), and subcortical regions were segmented using an automatic brain segmentation tool. Further details on image acquisition and preprocessing can be found elsewhere (Casey et al., 2018; Hagler et al., 2019).

We used diffusion spectrum images, which had undergone quality control according to a protocol (Hagler et al., 2019) by the ABCD Data Analysis and Informatics Center. With the quality‐controlled data, we performed the preprocessing procedure using MrTrix3 (Tournier et al., 2019) and FSL (Jenkinson, Beckmann, Behrens, Woolrich, & Smith, 2012). To analyze brain structural connectivity, we generated 84 × 84 whole‐brain individual connectomes using MRtrix3 software. See Appendix S2 for detailed descriptions of diffusion image preprocessing steps.

### Generalized random Forest and statistical analysis

We utilized GRF to examine the environmental, neural, and genetic variables underlying the individual differences in children's ADHD symptoms following stress exposure. Traditional mediation and moderation analyses, while effective for testing associations between treatment and outcome with a single variable, often fall short in unraveling nonlinear combinations of multiple covariates. The nonparametric machine‐learning approaches like GRF offer a more nuanced understanding of these sophisticated dynamics by modeling nonlinear, multivariate relationships. This capability is particularly beneficial in our context, where multiple factors are intricately intertwined. Therefore, we opted for GRF to uncover conditional patterns of treatment effect heterogeneity that might be overlooked by traditional linear models.

GRF ensures reliable estimation via training trees on one data subset and assessing treatment effects on a separate subset (Wager & Athey, 2018). It repeatedly splits the data, creating decision trees by maximizing treatment effect differences (Wiemken et al., 2021). This approach mitigates overfitting and enhances treatment effect reliability. Additionally, GRF reduces confounding bias by applying a doubly robust estimator (i.e. augmented inverse‐propensity weighting) when estimating the treatment effect (Glynn & Quinn, 2010).

GRF estimates both the average treatment effect (ATE) and the individual treatment effect (ITE) on the outcome. ATE measures the difference in the mean level of ADHD symptoms that would have been present with and without the treatment (i.e. stressful events) (Shiba et al., 2021). A significant ATE indicates that, on average, stress‐exposed children exhibit more ADHD symptoms than nonexposed children. ITE, on the other hand, provides information about individual‐level effects, highlighting individual differences. Calibration tests evaluate estimate validity, with model fit and heterogeneity indices close to 1 indicating good calibration and accurate treatment effect estimation (Athey & Wager, 2019).

#### Data preparation

Before training GRF models, we used baseline neuroimaging data to identify brain features associated with ADHD symptoms measured at baseline, 1‐year follow‐up, and 2‐year follow‐up. Separate regression models were run for each time point's ADHD outcome, controlling for sociodemographic factors and PRSs, to identify baseline neural markers predictive of both concurrent and future symptoms. This feature selection step yielded 29 baseline brain features significantly related to baseline ADHD symptoms and 3 baseline features each for the 1‐year and 2‐year follow‐ups (pFDR < .05; Table S5). These selected baseline features were then included as covariates in the corresponding GRF models. Specifically, including sociodemographic and genetic variables, we had a total of 71 covariates at baseline and 55 covariates at 1‐year and 2‐year follow‐ups. The abuse variable was omitted from the analyses due to its near‐zero variance (*p* > .05), indicating redundancy.

#### Analyses

Incorporating these covariates, we constructed GRF models to analyze the ATE and ITE of stressful events on ADHD symptoms at each time point. Still, due to the machine‐learning nature of GRF, treatment effects can vary based on seed number and covariate order. The large number of covariates may also weaken the estimation power (Athey & Wager, 2019), necessitating model optimization. Therefore, we conducted a three‐step analysis approach (i.e. first, second, and final model) (Figure 2).

The three‐step analysis enhances the reproducibility of results by (1) randomly changing the order of covariates three times, resulting in Random Iterations 1, 2, and 3, and then (2) using a seed ensemble technique. Instead of relying on one seed, the seed ensemble technique combines three forests built with different random seeds to make a big forest, improving result reliability.

Furthermore, the three‐step analysis helps identify the optimal model through feature (covariate) selection based on variable importance. The variable importance provides the estimate and ranking of variables based on their splitting frequency (Wang & Yang, 2022). Specifically, in the ‘*first model*’, we used all identified covariates when fitting the model. After fitting each model with different covariate orders and seed ensembles (i.e. Random Iterations 1, 2, and 3), only covariates above the mean importance estimate were retained. The intersection of these covariates across iterations formed the second covariate set. Using this covariate set, the ‘*second model*’ was fitted following the same procedure, resulting in a sparser set of final covariates. The ‘*final model*’ was then fitted with these covariates through repeated procedures (Figure 2). This optimization process narrows down the covariates to those that significantly contribute to individual differences, yielding three final models (i.e. Random Iterations 1, 2, and 3). We compared the results of three iterations to check the robustness. The significance and calibration of the models were assessed using the ATE estimate and model calibration test.

Based on our final model, we validated individual differences using the group average treatment effect (GATE) test (Athey & Wager, 2019). The GATE test stratifies the study sample into groups based on the predicted value of ITEs and compares the estimated ATE of these groups using a *t*‐test. We ranked the participants into three groups: low‐, middle‐, and high‐risk groups. A significant difference between groups confirms individual differences. A monotonic increase in estimated ATE across these groups is also critical, as it confirms that the treatment effect is lowest in the low‐risk group and highest in the high‐risk group.

As a final step, we applied our final model to compare covariate values between low‐ and high‐risk groups for ADHD symptoms, aiming to detect potential risk and protective factors. While this test effectively highlights group differences, its univariate nature limits its capacity to address nonadditive, conditional contributions of covariates. To overcome this, we also performed partial dependence simulations for the covariates (Friedman, 2001; Yi et al., 2024). For each covariate, we generated 100 synthetic samples, each representing various percentile values of that covariate while keeping other covariates fixed at their median values. These samples were input into the final model to observe how the simulated individual treatment effects varied with the covariate percentiles. An increase in treatment effect suggests that a covariate is likely a risk factor for the impact of stressful events on children's ADHD symptoms, while a decrease suggests a protective factor. This approach offers insights into how each covariate influences ADHD symptoms, considering multivariate interactions.

We used the R package ‘*grf*’ (v.2.1.0). All continuous variables were standardized (*z*‐scaled) prior to analysis. Also, since the ABCD data is recruited from 21 sites across the United States, we considered the site as a clustering factor to adjust its influence on the outcome measure. Family‐level clustering was not applied, as our genetic quality control procedure removed closely related individuals (e.g. siblings and relatives up to the third degree; see Appendix S2), leaving fewer than 0.5% of participants with a sibling in the sample. In addition, the GRF algorithm requires sufficiently sized clusters for stable resampling, and the small size of remaining family clusters would not allow meaningful clustering.

#### Specificity analyses

To confirm whether the individual differences discovered in our analyses are specific to ADHD symptoms, we additionally tested other mental disorders, including depression, anxiety disorder, somatic problems, oppositional defiant disorder (ODD), and conduct disorder. These mental disorders, like ADHD, were assessed using CBCL. For each disorder, we tested the ATE estimate, model calibration, and performed the GATE test. These analyses help determine if individual difference patterns observed in ADHD symptoms are unique or if similar patterns exist across other mental health outcomes.

## Results

Our three‐step model analysis culminated in a final optimized model that demonstrated an increase in ADHD symptoms following exposure to stressful events at the 1‐year and 2‐year follow‐ups (1‐year: ATE = 1.29; 2‐year: ATE = .94). Additionally, the final model revealed individual differences in these effects (1‐year: Heterogeneity index = 1.35; 2‐year: Heterogeneity index = 1.26) (Table 1). At baseline, the experience of stressful events was associated with increased ADHD symptoms (*p* < .05); however, the first model showed no significant variation among individuals (*p* > .05), eliminating the need for further testing (Table S6). The results of Random Iteration 1 are presented in Table 1, and the results of Random Iterations 2 and 3 are presented in Table S6.

**Table 1:** Final model (Random Iteration 1) results of average treatment effect and model calibration

	ATE	FDR	Model fit	FDR	Heterogeneity index	FDR
1‐Year follow‐up	1.29	2.0E‐09	.99	1.6E‐17	1.35	.006
2‐Year follow‐up	0.94	5.5E‐05	.90	2.2E‐08	1.26	2.0E‐05

A model fit and heterogeneity index close to 1 indicate a good fit. Detailed results of Random Iterations 2 and 3 are presented in Table S6. ATE, average treatment effect.

**Figure 3:**
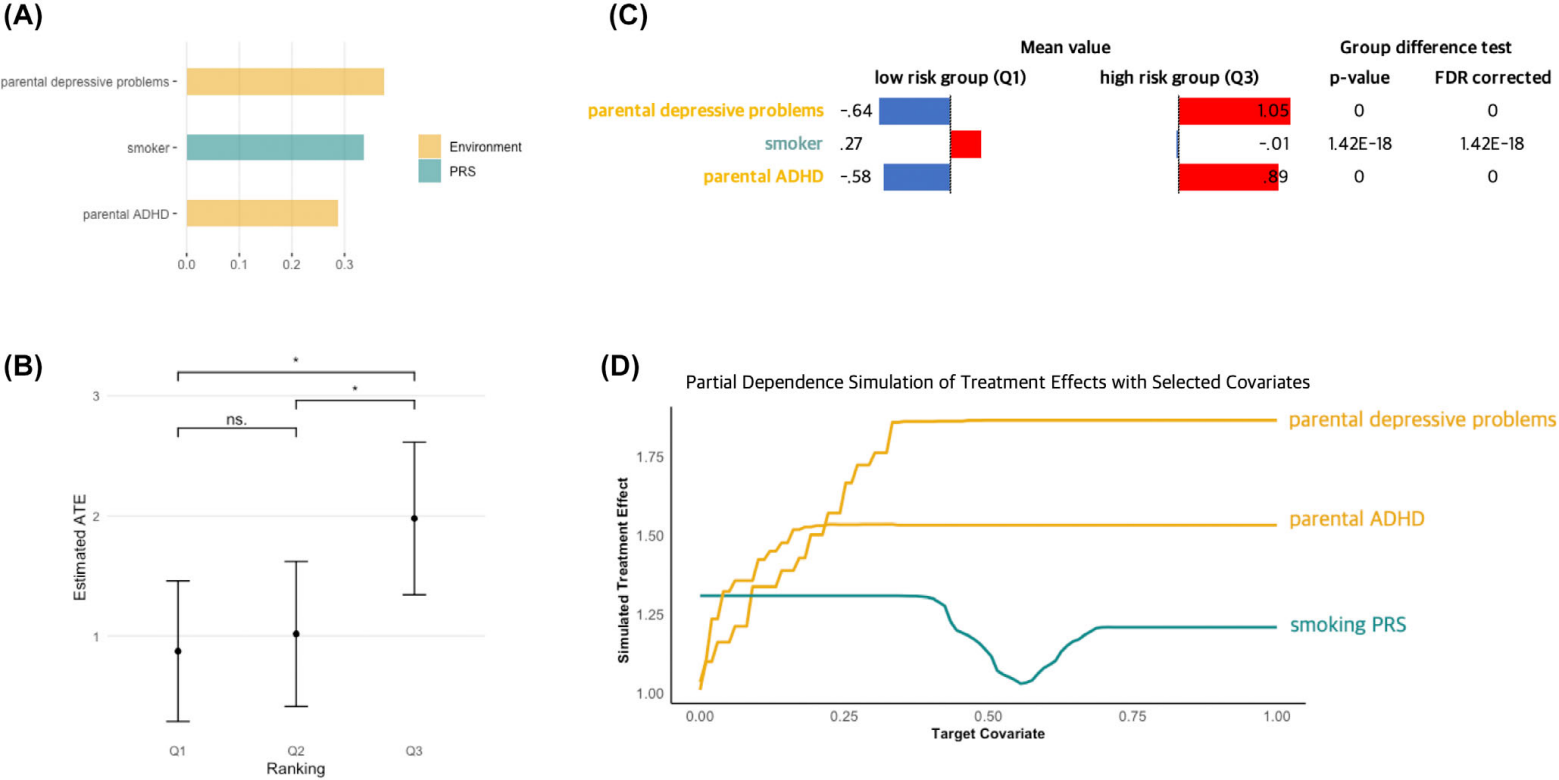
Final model (Random Iteration 1) results of the GATE test and covariate analyses at 1‐year follow‐up. (A) Variable importance results. The x‐axis refers to the variable importance estimate. The yellow bar indicates environmental features, and green indicates polygenic risk score. These three covariates were the variables that consistently showed above‐mean importance in the first and second models. (B) The GATE test results. The results showed significant group differences between the high‐risk and both the low‐ and middle‐risk groups, suggesting the presence of individual differences on ADHD symptoms at 1‐year follow‐up following early‐life stress (p FDR < .05). Q1 refers to the low‐risk group; Q2, the middle‐risk group; and Q3, the high‐risk group. (C) The comparison results of covariate values between the low‐ and high‐risk groups. All covariate values are z‐scaled. The high‐risk group showed more parental mental health problems but a lower polygenic risk score for smoking than the low‐risk group. (D) Partial dependence simulation results. The x‐axis refers to the covariate value, and y‐axis refers to the simulated treatment effect of each covariate while holding other covariates constant. The risk factors showed increasing treatment effect, whereas the protective factor exhibited decreasing treatment effect, reflecting its conditional contribution to individual differences in stress‐related ADHD symptom vulnerability. Detailed results for all Random Iterations are presented in Figures S1 and S2; Table S7. ATE, average treatment effect; GATE, group average treatment effect; PRS, polygenic risk score; ns, not significant; *** *p* < .001; **p < .01; *p < .05

At the 1‐year follow‐up, each stage of our three‐step modeling process yielded significant results (*p* < .05; Table S6), resulting in a final model with three key covariates (i.e. parental depressive problems, parental ADHD, and smoker PRS) (Figure 3A) that showed consistent importance in the first and second step models (Figure S1). The GATE test revealed significant differences between the low‐ and high‐risk groups in the final model (low‐risk (Q1): ATE = .87; high‐risk (Q3): ATE = 1.98; Q3–Q1: p FDR < .05) (Figure 3B, Table S7), validating the individual differences. High‐risk children exhibited higher levels of parental depressive problems and parental ADHD but lower levels of polygenic scores for smoking compared to low‐risk children (Figure 3C, Figure S2). The partial dependence simulation confirmed this pattern, showing an increasing treatment effect for vulnerable factors (i.e. parental depressive problems and parental ADHD) and a decreasing effect for the resilient factor (i.e. smoking PRS) (Figure 3D, Figure S2).

**Figure 4:**
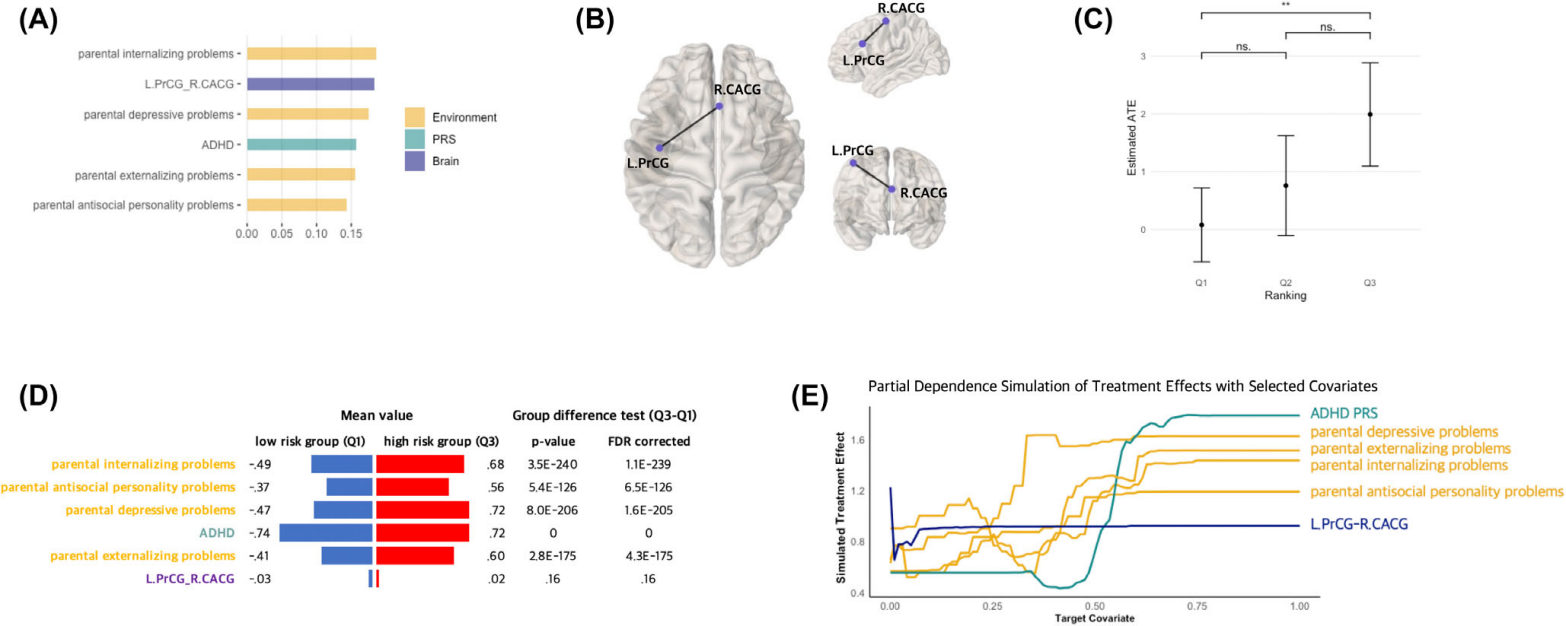
Final model (Random Iteration 1) results of the GATE test and covariate analyses at 2‐year follow‐up. (A) Variable importance results. The yellow bar indicates environmental features, green for polygenic risk score, and blue for brain features. These six covariates were the variables that consistently showed above‐mean importance in the first and second models. (B) The structural connectivity between the left precentral gyrus (PrCG) and right caudal anterior cingulate gyrus (CACG). This connectivity was included as a covariate in the 2‐year follow‐up models. (C) The GATE test results. The results showed significant group differences between the low‐ and high‐risk groups, suggesting the presence of individual differences in ADHD symptoms at 2‐year follow‐up following early‐life stress (p FDR < .05). Q1 refers to the low‐risk group; Q2, the middle‐risk group; and Q3, the high‐risk group. (D) The comparison results of covariate values between the low and high‐risk groups. All covariate values are *z*‐scaled. The high‐risk group showed more parental mental health problems and a higher polygenic risk score for ADHD than the low‐risk group. (E) Partial dependence simulation results. The x‐axis refers to the covariate value, and the y‐axis refers to the simulated treatment effect of each covariate. An increase in simulated treatment effect was found for most risk factors; however, the streamline count between left PrCG and right CACG showed a slight initial increase but remained constant. Detailed outcomes for all Random Iterations are presented in Table S8, Figures S3 and S4. L.PrCG, left precentral gyrus; R.CACG, right caudal anterior cingulate gyrus; ATE, average treatment effect; GATE, group average treatment effect; PRS, polygenic risk score; ns, not significant; ***p < .001; **p < .01; *p < .05

At the 2‐year follow‐up, out of 55 initial covariates, the final model included six key covariates: parental internalizing problems, parental externalizing problems, parental depressive problems, parental antisocial personality problems, ADHD PRS, and streamline count between left precentral gyrus (PrCG) and right caudal anterior cingulate gyrus (CACG) (Figure 4A, Figure S3). This final model demonstrated robustness through the GATE test, showing significant differences between the low and high‐risk groups across all three Random Iterations (low risk(Q1): ATE = .08; high risk(Q2): ATE = 1.99; Q3–Q1: p FDR < .01) (Figure 4C, Table S8).

In assessing group differences in covariates, the high‐risk group exhibited more parental mental health issues and a higher PRS for ADHD than the low‐risk group (p FDR < .05) (Figure 4D). However, the streamline count between the left PrCG and the right CACG varied across iterations (Figure S4). The partial dependence simulation showed a similar pattern. The treatment effect increased with higher ADHD PRS and parental mental illnesses (i.e. depressive, externalizing, internalizing, and antisocial personality problems) (Figure 4E, Figure S4). However, the streamline count between the left PrCG and right CACG showed a slight initial increase but then remained constant, reporting the lowest treatment effect among the covariates. This finding may imply that the neural factor's contribution to individual differences might not be as robust as other covariates, such as parental mental health problems and ADHD PRS.

Although sex did not emerge as a top‐ranked covariate contributing to individual differences in the main GRF models, we conducted additional sex‐stratified analyses to explore potential sex‐specific patterns of vulnerability, given the well‐documented sex differences in ADHD (Arnett, Pennington, Willcutt, DeFries, & Olson, 2015) (Appendix S3). We found that the impact of stressful life events was significantly associated with ADHD symptoms over time in both males and females (Tables S9 and S10). However, significant individual variability in treatment effects was observed only in the male group at the 2‐year follow‐up, as indicated by a significant heterogeneity index (p FDR < .05) (Table S9).

The final optimized model in the male sample at 2‐year follow‐up identified five key covariates contributing to individual differences in stress‐related ADHD outcomes: parental depressive problems, parental internalizing problems, parental externalizing problems, ADHD PRS, and streamline count between the right inferior parietal gyrus (IPG) and right posterior cingulate gyrus (PCG) (Figure S5). This model demonstrated robustness in the GATE test, showing significant differences in estimated treatment effects between low‐ and high‐risk groups (Figure S6). High‐risk boys were characterized by elevated parental mental health problems, higher ADHD PRS, and greater white matter streamline count between the right IPG and right PCG. However, similar to the main 2‐year model, the neural feature varied across model iterations, suggesting a less robust contribution of neural factors compared to familial and genetic variables in explaining individual differences.

### Specificity analysis

To test whether our findings of individual differences in the impact of stressful events are specific to ADHD symptoms, we applied the final model of 1‐year and 2‐year follow‐ups to other mental disorder outcomes (i.e. depression, anxiety disorder, somatic problems, ODD, and conduct disorder). We conducted the GATE test for models showing both significant model fit and heterogeneity index: anxiety disorder and conduct disorder at 1‐year follow‐up, and depression, anxiety disorder, and ODD at 2‐year follow‐up (*p* < .05) (Table S11). As a result, these five models revealed no significant GATE results (Figure S7), underscoring the specificity of our model to ADHD symptoms.

While some significant group differences were observed in the model with conduct disorder at 1‐year follow‐up, and with anxiety disorder and ODD at 2‐year follow‐up the estimated ATE of the three groups did not show a consistent increase. This lack of monotonic increase indicates that the causal forest predictions did not align with the estimated ATEs, showing the specificity of our model to ADHD symptoms.

## Discussion

This study offers new insights into the multifaceted determinants of ADHD symptoms following stressful life events, highlighting the significance of parental influence, genetic predisposition, and neural factors in shaping children's susceptibility or resilience. Notable individual differences emerged in ADHD symptom progression at 1‐year and 2‐year follow‐ups, with such variations absent at baseline, suggesting the evolution of vulnerability or resilience factors over time. Our comprehensive assessment of environmental, genetic, and neural factors identified key contributors to these differences, underscoring the importance of personalized approaches in both research and clinical practice.

Initially, the impacts of stressful events on ADHD symptoms did not vary significantly among children; however, with time, marked individual differences became apparent. This trend indicates that children's initial resilience to stress may not exert a strong influence on subsequent symptomatology, but as time progresses, these factors increasingly affect symptom trajectories, with certain children exhibiting heightened vulnerability. Literature supports this notion, demonstrating that the mental health outcomes for vulnerable children tend to deteriorate, while resilient children's conditions typically stabilize or improve (Masten & Cicchetti, 2010; Masten & Tellegen, 2012).

As our findings indicate, the divergence in response to stress pertaining to ADHD symptoms may result from the combined influence of neural, behavioral, and genetic factors. At the 1‐year follow‐up, children who were more susceptible to exhibiting increased ADHD symptoms upon exposure to stress were identified by the nonlinear patterns among several factors: higher instances of parental depressive problems and ADHD and a lower polygenic score of smoking. Such elevated parental mental health challenges persisted into the 2‐year follow‐up, suggesting their enduring influence on symptom development. Parental psychopathology may heighten vulnerability by creating a chronically challenging or less supportive family environment for the child. Prior studies have shown that such negative family environments contribute to persistent psychological distress and shape long‐term developmental trajectories (Cobham, Brett, Divna, & Sanders, 2016; Kamis, 2021).

Surprisingly, a lower polygenic score for smoking emerged as a risk factor at 1‐year follow‐up, a finding that defies conventional expectations. While speculative, this may reflect complex multivariate relationships uniquely captured by the GRF model. By employing GRF, our study was able to uncover complex relationships among genetic, neural, and environmental factors that may be obscured when focusing on isolated predictors. For instance, whereas previous regression‐based studies identified smoking‐related PRS as markers of psychiatric risk (Jansen et al., 2021), our GRF approach surfaced a counterintuitive vulnerability signal in the context of early‐life stress and ADHD symptom development. Supporting this interpretation, another study using the GRF methodology uncovered distinct patterns of susceptibility to post‐traumatic stress symptoms, including factors like higher education levels, lower family income, and significant depressive issues (Shiba et al., 2022). These findings collectively underscore the value of multivariate analytical approaches in identifying nonobvious, conditional predictors to fully understand the intricate dynamics of ADHD symptomatology following early‐life stress.

By the 2‐year follow‐up, the vulnerability pattern shifted, encompassing a higher ADHD PRS, exacerbated parental mental health issues, and increased neural connectivity, particularly a greater white matter streamline count between the left PrCG and right CACG measured at baseline. This connection is part of the cognitive control network (CCN), which is crucial for attention, working memory, and executive functions (Cole & Schneider, 2007). Disrupted functional connectivity in the CCN has been reported in trauma‐exposed veterans (Kennis, Rademaker, Van Rooij, Kahn, & Geuze, 2015) and ADHD adolescents (Francx et al., 2015). However, our results showed that the streamline count between the left PrCG and right CACG was not an effective risk factor compared to other covariates. This may be due to the neurodevelopmental processes during adolescence, as higher‐order cognitive functions like executive function continue to mature into adulthood (Best & Miller, 2010). Given that the mean age of participants in our study at baseline was 9.9 years, their structural connectivity between PrCG and CACG may still be maturing, making the differences between low and high‐risk children less robust.

Although many children exhibit stress resilience by maintaining a capacity to buffer adversity's impacts (Alvord & Grados, 2005; Zolkoski & Bullock, 2012), there are children who remain at heightened risk. Thus, the gene‐brain‐environmental patterns identified at the 2‐year follow‐up could be critical markers of sustained vulnerability, reflecting an enduring susceptibility to ADHD symptoms over time. Unraveling how these intricate relationships evolve over time could lead to the development of more effective, personalized strategies to support children facing early‐life stress and to prevent the exacerbation of ADHD symptoms.

The complex, nonlinear associations between PRS (i.e. smoking, ADHD), brain structural connectivity, and environmental factors (i.e. parental mental illness) in the association between early‐life stress and ADHD symptoms may be explained by epigenetic modifications highlighted in a prior study (Niwa et al., 2013). The study demonstrated that stress during critical developmental periods, such as adolescence, can induce lasting behavioral and neurobiological changes via glucocorticoid‐mediated epigenetic control of dopaminergic neurons when combined with specific genetic risks (Niwa et al., 2013). Using a mouse model, they found that elevated glucocorticoids led to significant changes in dopaminergic projections from the ventral tegmental area only in the presence of both genetic predispositions and environmental stressors, highlighting a crucial interplay between genes and environment. These dopaminergic projections are involved in numerous brain functions, such as cognitive control and motivation (Cools, 2016), which are highly related to ADHD (Swanson et al., 2007). Thus, variability in ADHD symptom progression among children following stressful events can be partially explained by these findings. Indeed, a review on ADHD reported the associations with ADHD and dopamine genes, abnormal structure and function in brain regions related to dopamine (Swanson et al., 2007), along with significant effects of dopamine genes on brain anatomy (Durston, Mulder, Casey, Ziermans, & van Engeland, 2006). Due to the lack of physiological data in the ABCD study, further study is necessary to disentangle the relationships between genes, brain, and environment from a molecular perspective, which could lead to more personalized approaches in managing ADHD symptoms after stress exposure.

Sex‐stratified GRF analyses revealed that stressful events significantly impacted ADHD symptoms in both sexes from baseline to the 2‐year follow‐up. However, individual variability was observed only in males at follow‐up, suggesting greater heterogeneity in stress‐related ADHD symptom trajectories among males. While parental mental health and ADHD polygenic risk were common contributors to this heterogeneity across models, the neural feature identified in the male‐specific model (right IPG–right PCG structural connectivity) differed from the whole‐sample model (left PrCG–right CACG structural connectivity). The PrCG‐CACG pathway, connecting motor and cognitive control regions, may reflect a general pathway for effortful control or response inhibition, broadly relevant across sexes. In contrast, the IPG‐PCG connectivity may reflect a male‐specific neural vulnerability to stress‐related ADHD development. This interpretation is supported by studies linking altered IPG–PCG connectivity with hyperactivity and attentional problems in hyperthyroidism (Tesfaye, Getnet, Bitew, Adugna, & Maru, 2024), symptoms more prevalent in males.

The absence of the PrCG–CACG connection in the male‐specific model may indicate that, while broadly relevant, it is less salient when sex‐specific profiles are considered. Conversely, the IPG‐PCG connection may be a more male‐predominant vulnerability marker that becomes prominent only when male‐specific patterns are not diluted by other factors in the combined group analyses. Nonetheless, it is important to interpret these findings with caution, as neural features showed less robustness and consistency across iterations compared to genetic and familial factors. Future study using clinical ADHD samples across sexes is needed to clarify the neural mechanisms underlying sex‐specific vulnerability and resilience to stress‐related ADHD.

This study marks a significant advancement in understanding how early‐life stress contributes to ADHD symptom development by revealing individual‐level heterogeneity in children's responses to stress. Utilizing GRF, a flexible nonparametric algorithm, we move beyond traditional models that focus on average effects and demonstrate that stress‐related symptom trajectories vary based on each child's unique combination of genetic, neural, and familial risk factors. Key contributors to this heterogeneity include parental psychopathology, ADHD PRS, and specific brain connectivity features. Clinically, these findings highlight the importance of multimodal risk profiling over reliance on single predictors. Children exposed to early‐life stress who also present with high parental psychopathology or elevated genetic risk may benefit from proactive monitoring and early, targeted interventions. Rather than relying solely on categorical diagnoses or symptom thresholds, clinicians could leverage individualized risk profiles to guide preventive strategies, such as family‐based interventions or behavioral parent training.

The identification of time‐varying predictors further underscores the need for developmentally sensitive approaches. Notably, heterogeneity in ADHD outcomes became more pronounced by the 2‐year follow‐up, suggesting early adolescence (~12 years in this cohort) as a critical window when stress‐related vulnerability becomes more apparent. This finding supports the value of longitudinal screening and adaptive intervention models that evolve with the child's developmental trajectory, rather than relying on static, one‐time assessments.

Still, there are limitations to consider. Our operational definition of stressful life events, based on exposure, may not adequately capture the subjective intensity of these experiences, pointing to a direction for future research. This choice was necessitated by the study design, which required a baseline measure of stress exposure, and by data availability in the ABCD study, where subjective measures of stressful events (e.g. Youth Life Events) were not collected at baseline but introduced in later follow‐up assessments. Future research incorporating subjective appraisals of stress would be necessary to gain a more complete understanding of individual variability in the impact of adversity. Additionally, GRF results might vary with changes in seed numbers or the ordering of covariates. We addressed this by performing multiple iterations with different seed numbers and sequences of covariates, complemented by a three‐step model analysis to ensure robustness (i.e. first model, second model, and final model). Lastly, the study's scope, while comprehensive, does not extend into the potential long‐term impacts of early‐life stress into adolescence and adulthood, which warrant further investigation.

## Ethical considerations

All study procedures were approved by the centralized institutional review board (IRB) at the University of California, San Diego (IRB# 160091, approved September 13, 2016), as well as by the local IRBs at all 21 data collection sites. Written informed consent was obtained from the parents or legal guardians, and the child participants provided written assent.

**Key points**

**What's known?**

- Previous studies have shown that stressful life events can exacerbate ADHD symptoms. However, most research has focused on average group‐level effects, overlooking individual heterogeneity in stress responses.

**What's new?**

- This study employs a novel nonparametric machine‐learning approach to identify key factors that influence whether a child becomes more vulnerable to or resilient against developing ADHD after experiencing stress. These factors include parental mental health issues, ADHD polygenic risk scores (PRS), and brain connectivity patterns, which showed different patterns as children age.

**What's relevant?**

- Identifying these key risk factors enables clinicians to tailor interventions specifically to children at higher risk for developing ADHD following stress exposure. The research emphasizes the importance of considering complex, conditional relationships among genetic, neural, and environmental factors in future ADHD and stress studies, advocating for more sophisticated analytical approaches to uncover these intricate dynamics.

## Supporting information


**Appendix S1.** Multiethnic PRS results.
**Table S1.** Demographic of the multi‐ancestry sample at baseline and follow‐up periods.
**Table S2.** The three‐step model results in the impact of stressful events on ADHD symptoms at baseline, 1‐year follow‐up, and 2‐year follow‐up in multi‐ancestry sample.
**Table S3.** Demographic of the sample at baseline and follow‐up time points.
**Appendix S2.** Covariate measurements.
**Table S4.** Genome‐wide association studies used for polygenic risk score generation.
**Table S5.** The results of regression analyses to extract ADHD‐specific brain features.
**Table S6.** The three‐step model results in the impact of stressful events on ADHD symptoms at baseline, 1‐year follow‐up, and 2‐year follow‐up.
**Figure S1.** Variable importance of three‐step models in the impact of stressful events on ADHD symptoms at 1‐year follow‐up.
**Table S7.** The GATE test results of final model evaluating the impact of stressful events on ADHD symptoms at 1‐year follow‐up.
**Figure S2.** The results of group difference test and partial dependence simulation at 1‐year follow‐up.
**Figure S3.** Variable importance of three‐step models in the impact of stressful events on ADHD symptoms at 2‐year follow‐up.
**Table S8.** The GATE test results of final model evaluating the impact of stressful events on ADHD symptoms at 2‐year follow‐up.
**Figure S4.** The results of group difference test and partial dependence simulation at 2‐year follow‐up.
**Appendix S3.** Sex‐stratified GRF analyses.
**Table S9.** The results of sex‐stratified GRF analysis in male group.
**Table S10.** The results of sex‐stratified GRF analysis in female group.
**Figure S5.** Variable importance of three‐step models in the impact of stressful events on ADHD symptoms at 2‐year follow‐up in male group.
**Figure S6.** The GATE test, group difference test, and partial dependence simulation results of the final model evaluating the impact of stressful events on ADHD symptoms at 2‐year follow‐up in male group.
**Table S11.** The final model results of specificity analyses in the impact of stressful events on different mental disorders at 1‐year and 2‐year follow‐ups.
**Figure S7.** The GATE test of specificity analyses in the impact of stressful events on different mental disorders at 1‐year and 2‐year follow‐ups.

## Data Availability

Data were obtained from the ABCD study (http://abcdstudy.org). To access, users are required to register for an account via the NIMH Data Archive and adhere to the instructions provided on the website to secure permission. GRF algorithm code can be found at https://grf‐labs.github.io/grf/index.html.
